# Body Image and Body Avoidance Nine Years After Bariatric Surgery and Conventional Weight Loss Treatment

**DOI:** 10.3389/fpsyt.2019.00945

**Published:** 2020-01-14

**Authors:** Tanja Legenbauer, Astrid Müller, Martina de Zwaan, Stephan Herpertz

**Affiliations:** ^1^Department for Child and Adolescent Psychiatry, Psychosomatic and Psychotherapy, LWL University Hospital of the Ruhr-University Bochum, Hamm, Germany; ^2^Department of Psychosomatic Medicine and Psychotherapy, Hannover Medical School, Hannover, Germany; ^3^Department of Psychosomatic Medicine and Psychotherapy, LWL University Hospital of the Ruhr-University Bochum, Bochum, Germany

**Keywords:** obesity, bariatric surgery, body image, body avoidance, weight loss, eating pathology

## Abstract

Recently, there has been an increasing focus on body image dissatisfaction (BID), both as a motivational factor for seeking bariatric surgery and as a factor influencing weight loss outcome after surgery. Although associations have been reported between BID, emotional distress and successful weight loss, conclusions are limited due to methodological issues such as non-weight-specific assessment tools for body image and neglect of behavioral components (e.g. body avoidance, BA). The present study seeks to report on BID and BA 9 years after bariatric surgery using a cross-sectional data set from the 9-year follow-up assessment of the Essen–Bochum Obesity Treatment Study (EBOTS). In total, N = 291 participants of the original EBOTS sample were included in the present analyses (N = 78 bariatric surgery patients, SURG; N = 124 patients of a conventional treatment program, CONV; and N = 83 individuals with obesity not seeking treatment, OC). Current body image facets (BID and BA) were captured at the 9-year follow-up assessment *via* silhouette scales adapted for use in samples with obesity. Moreover, BID was assessed retrospectively to obtain baseline attitudes. Possible influences of eating disorder symptoms and depression/anxiety were controlled for and assessed *via* standardized self-report measures. The results imply an improvement in BID in the SURG group, but not in the CONV and OC groups. The level of BA in relation to clothing was significantly higher in the CONV group compared to both the SURG and OC group. Current BID as well as BA were positively associated with current body weight as well as depression, anxiety, and levels of disinhibited eating. A positive change from baseline to current levels of BID was associated with successful weight loss, independently of treatment. The findings emphasize the role of the different components of body image after surgery for mental health features, and suggest a robust relationship between BID and weight loss (success). Thus, it might be helpful to address BID in treatment. However, further research, particularly in the form of prospective studies, is necessary to determine the direction of influence.

## Introduction

Obesity [defined as a body mass index (BMI) ≥30 kg/m^2^] is a clinical disease with a high risk of chronicity. As such, prevention and management of obesity have become important public health issues. Bariatric surgery has received increased attention, in particular for the treatment of individuals with severe obesity, as it successfully reduces weight, with major physiological benefits also in the long term (1). However, reports regarding mental health and course of weight after surgery have shown less favorable outcomes in some patients (2–5). In this regard, research has recently begun to focus on body image dissatisfaction, both as a motivational factor for seeking bariatric surgery (6, 7) and as a factor influencing weight loss outcome after surgery (8).

Body image needs to be considered as a multifaceted construct, which encompasses perceptual deficits (e.g. seeing oneself as fatter than one is), cognitive-affective/attitude distortion (e.g. thinking negatively about one’s body), and dysfunctional body-related behaviors such as checking and avoidance behaviors [e.g. (9–11)]. Body image disturbances are common among young people, and especially among females [e.g. (12)], and are core symptoms of eating disorders such as anorexia or bulimia nervosa (13). However, over the last decade, robust evidence has emerged that body image disturbances also occur in individuals with obesity. Study findings have emphasized associations between obesity and cognitive/attitudinal components of body image, indicating a relation between higher dissatisfaction/low appearance evaluation and increasing weight. Moreover, heightened body avoidance and checking behaviors are also common among this patient group [e.g. (8, 14, 15)]. There is also some evidence regarding the misperception of body size (14, 16), as well as robust evidence that body image dissatisfaction (BID) in individuals with obesity seeking bariatric surgery is related to increased distress (17). In particular, depression, anxiety, and suicidality have been associated with BID pre-surgery, and interestingly, emotional eating served as a mediator between BID and psychological stress (18).

Given that a negative body image is a motivational factor to undertake weight loss efforts and is related to the level of physical activity, dietary control strategies, and caloric intake, etc. (14), changes in body weight might be associated with enhanced body image. Indeed, several studies showed improvements in body dissatisfaction after conventional, non-surgical weight loss treatments that were associated with weight loss outcome [e.g. (19–21)]. Moreover, in samples of individuals with severe obesity undergoing bariatric surgery, a positive body evaluation after bariatric surgery was associated with greater weight loss (22, 23) and better quality of life (8), whereas poor weight loss outcome after gastric banding was associated with persistently negative body image (24). A negative body perception following surgery was associated with the presence of psychopathology, such as higher depression or anxiety (8).

Despite this preliminary evidence supporting an association between body image and weight change after conventional and surgical weight loss treatment, some methodological issues have to be considered, which probably limit the generalizability of these findings: For instance, most studies relied on non-weight-sensitive assessment tools such as questionnaires that were developed and validated within the normal-weight general population and thus do not consider differences in attitudes and experiences between normal-weight and individuals with obesity (25, 26). Furthermore, most studies did not control for eating disorder symptomatology, although it has been shown that the presence of an eating disorder is linked to greater BID among individuals with obesity (16). In addition, most studies neglected the multifaceted nature of body image by focusing only on cognitive-attitudinal components (27). As behavioral aspects of body image, such as avoidance and checking behaviors, are known to maintain a negative body image and are also related to eating pathology in bariatric surgery patients (28), it seems important to include these components when investigating weight loss outcome and body image in individuals with obesity seeking bariatric surgery. However, to our knowledge, there is only one recent study that included body avoidance and checking behavior besides attitudinal aspects in a pre- to 6-month post-surgery assessment (29). The results showed that body dissatisfaction, feelings of fatness, and body avoidance were significantly reduced 6 months after surgery, with the largest reduction being found for body avoidance.

In summary, body image features seem to be associated with the course of weight after bariatric surgery and might be accompanied by eating disturbances (emotional eating) and psychological distress (e.g. higher depression or anxiety scores). Current evidence is limited to questionnaire-based, non-weight-sensitive assessments and mostly neglects the multifaceted nature of body image. Moreover, comparisons between conventional and surgical procedures for weight loss and non-treatment-seeking individuals with obesity, as well as investigations with long-term outcome monitoring, are lacking. Consequently, further research is warranted to better understand the associations between body image and its different facets, and the changes therein following bariatric surgery. The present study therefore seeks to report on body image attitudes and behaviors 9 years after surgery using a cross-sectional data set from a 9-year follow-up assessment during the Essen–Bochum Obesity Treatment Study (EBOTS). A conventional treatment group as well as individuals with obesity who did not seek weight loss treatment acted as control groups. As the weight loss should be higher in those who underwent surgery, we assume a greater reduction in perceived body size and body dissatisfaction and lower avoidance-related behaviors in this group compared to both control groups. We also assume that the reduction in perceived body, body dissatisfaction, and body-related avoidance behavior will be associated with the amount of weight loss. Finally, we seek to explore the differential impacts of these components on successful weight loss while considering factors that might also be related to body image features and weight loss (eating disorder symptoms, depression and anxiety levels).

## Method

### Design and Procedure

The present cross-sectional analysis is part of a large controlled multicenter study (initiated in 2000) that aimed to prospectively investigate predictors of the short- and long-term course of weight after surgical and non-surgical weight-loss treatment. As the main research questions as well as detailed information concerning design and procedure have been published elsewhere (4, 30–38), only the most important information is summarized here: In 2000, the study began with a cross-sectional assessment comprising individuals with obesity seeking non-surgical and surgical weight loss treatment as well as controls with obesity. The control group was originally recruited from a random selection of the residents list (all citizens in Germany are legally required to register their place of residence) of the city of Essen (about 600,000 inhabitants) and matched for age and weight. Bariatric surgery patients were recruited from six surgery departments in Germany and were assessed on the day of hospital admission. Participants in the conventional treatment group had undergone the Optifast® program which included a multidimensional therapy approach (nutritional counseling, behavioral modification) with weekly group sessions over 1 year. During the initial 12 weeks of Optifast®, a liquid meal replacement was applied. The following exclusion criteria were applied for all participants: pregnancy, chronic, non-obesity-associated diseases or disabilities, or a diagnosis of psychotic disorder or dementia. In addition, participants of the population-based control group with obesity who reported that they were currently trying to lose weight were excluded.

All participants with obesity were approached at five assessment time points (baseline, 1, 2, 4, and 9 years after the intervention). At the 9-year assessment, participants were contacted by telephone and, after providing informed consent, were sent the self-report questionnaires. In addition, they either came to the treatment center or were visited at home to be interviewed with two clinical structured interviews by one of four trained clinical psychologists, who were monitored throughout the study. Body weight and height were measured under controlled conditions after the removal of shoes and heavy clothing. At the 9-year assessment, all participants received a reimbursement (120€). For the present study, data from both treatment groups as well as the control group with obesity were analyzed if data on body image variables assessed at the 9-year follow-up were available.

### Participants

Of the original baseline sample (n = 529), 55% of the participants took part in the 9-year follow-up assessment and provided information on body image (N = 291). Of these, N = 78 participants belonged to the *Bariatric surgery group* [SURG; 51% of the original baseline sample (N = 152)]. Most of the participants in this group had received restrictive procedures such as vertical gastroplasty or gastric banding. In total, 35% reported reoperations following the initial surgery in 2000.

N = 130 participants of the *Conventional treatment group* (CONV) provided the relevant questionnaires for the present analyses. Of these, six participants reported bariatric surgery before the 9-year follow-up assessment and therefore had to be excluded from the analyses; thus, N = 124 data sets were available for the CONV group [50% of the original baseline sample (N = 249)].

N = 83 participants with *obesity* of the *control group* (OC, individuals with obesity not initially seeking treatment) could be included in the analyses, corresponding to 68% of the original sample.

Reasons for dropouts were related to health problems (chronic illness) or death, pregnancy, or contact unknown, no response/refusal to participate. Differences between those who dropped out and those who participated at the 9-year follow-up related to relationship status and BMI, with dropouts being more likely be single and to have a lower baseline BMI. No further differences on clinical and sociodemographic values were found. A detailed overview of reasons for dropout and detailed sample characteristics was provided in (4).

### Ethics Statement

The study was approved by the ethics committee of the ethical board of the Medical Faculty of the Ruhr-University Bochum. The study protocol was conducted in accordance with the Declaration of Helsinki (revised 1983). Written informed consent was provided by all participants, who were aware that they could withdraw from the experiment at any time without further consequences.

### Assessment

The following areas of interest/assessment instruments applied at the 9-year follow-up were incorporated in the present analyses: 1) Body image was assessed with the Body Image Assessment for Obesity (BIA-O) and the Body Image Avoidance Questionnaire (BIAQ); 2) Eating pathology was assessed with the short version of the Structured Interview for Anorexic and Bulimic Disorders (SIAB-EX) and the Three-Factor Eating Questionnaire (TFEQ); and 3) General psychopathology was assessed with the Hospital Anxiety and Depression Scale (HADS). In addition, clinically relevant mental disorders were assessed with structured clinical interviews.

#### Body Image Assessment for Obesity

To measure body image in overweight adults, silhouette scales adapted for use among individuals with obesity (39) were applied. These silhouettes include the nine body silhouettes from the original version of this last scale, which range from underweight to overweight, plus nine body silhouettes that reflect different degrees of overweight to extreme obesity, in total 18 increments. Participants rated which silhouette corresponds best to their current body shape, thus representing the perceptual component of body image. This is labeled as “perceived shape” throughout the manuscript. Next, participants were asked to choose the silhouette which best reflects the shape they realistically would like to achieve in the future. This is labeled as “desired shape”. Finally, participants were asked to rate the silhouette that represents their ideal shape. The results for this last scale are reported in the **Supplementary Materials** see **Table S1**. In the current study, the three ratings had to be performed twice: a) retrospectively concerning the shape before the start of the weight loss intervention (baseline), and b) concerning the current shape. In addition, a discrepancy score indicating the degree of body dissatisfaction was calculated by subtracting the perceived shape from the desired shape.

#### Body Image Avoidance Questionnaire

The 11 items of the German version of the BIAQ (40) measure body-related avoidance behavior within the dimensions “clothing”, “social activities”, and “eating-related control behavior”. The items were rated on a 5-point scale from “not at all” (= 0) to “always” (= 4).

#### Three-Factor Eating Questionnaire

The German version of the Three-Factor Eating Questionnaire [TFEQ, (41)] was applied. The TFEQ measures behavioral correlates of dysfunctional eating along the dimensions “cognitive restraint”, “disinhibition”, and “feelings of hunger”. In the present analyses, only the disinhibition subscale was considered.

#### Hospital Anxiety and Depression Scale

The German version of the Hospital Anxiety and Depression Scale [HADS, (42)] was administered to assess anxiety and depressive symptoms. A score of >10 indicates clinically relevant symptoms.

#### *Composite International Diagnostic Interview*, M-CIDI/DIA-X

To assess the prevalence of mental disorders 9 years after treatment, the CIDI (43) was applied. The CIDI is a reliable structured clinical interview based on criteria of ICD-10 and DSM-IV. “Current diagnosis” refers to symptoms reported for the past 2 to 4 weeks. Reliability and validity of the M-CIDI/DIA-X have been confirmed in several investigations.

#### Structured Interview for Anorexic and Bulimic Disorders

The short version of the SIAB-EX (44) was used to assess eating disorder symptoms and diagnoses according to DSM-IV criteria. The reliability and validity of the structured interview in patients with eating disorders is well documented (44). Data from the SIAB in the present analyses concern the assessment of binge eating disorder as well as the presence of objective binge eating.

#### Sociodemographic characteristics and weight

Sociodemographic information was provided by all participants, and weight and height were assessed in light clothing without shoes. Successful weight loss (maintenance) was defined as the maintenance of at least 5% weight loss from the baseline weight. The percentage weight loss was calculated as baseline weight minus weight at 9-year follow-up divided by baseline weight, and the result was multiplied by 100: [KG(Base) − KG(9y)/KG(Base)] × 100. Then, a nominal variable was created to evaluate whether the percentage weight loss was lower or higher than 5%.

As definitions for successful weight loss for surgery patients differ markedly from those applicable for the conventional weight loss treatments, in line with the recommendations, the successful weight loss variable for the SURG group was created based on percent excess BMI loss {%EBMIL = [change in BMI/(Initial BMI − 25)] × 100}, an equivalent to %EWL according to the definition of the American Society for Metabolic and Bariatric Surgery [ASMBS (45)]. For those in the surgery group, 50% EBMIL is considered as representing a successful amount of weight loss (46). If this criterion was fulfilled, the participant was categorized as successful weight loser. We then collapsed the information on successful weight loss for both groups and created a single variable (successful weight loss) that reflects information whether a participant was categorized as successful weight loser 9 years after surgery or conventional weight loss treatment respectively.

#### Statistical Analyses

All analyses were performed using IBM^®^ SPSS version 24. Differences between all three groups regarding baseline characteristics were calculated using univariate or multivariate ANOVAs or χ^2^ tests. Changes in body image perception and attitudes assessed with the BIA-O were analyzed by repeated measures analyses of covariance with sex and age as covariates. A power analysis using G*Power3 (47) to test the difference in change between the three groups from baseline to 9-year follow-up with repeated measures ANCOVA assuming a medium effect size (f = 0.25), and an alpha of 0.05, revealed that a total sample of 66 participants was required to achieve a power of 0.95. Group differences in avoidance behavior (BIAQ subscales) were calculated using multivariate analyses of covariance, taking into account the influence of sex and age. Partial eta square (η_p_^2^) is reported to evaluate effect sizes of the results. In general, η_p_^2^ is the ratio of variance associated with an effect, taking into account the effect itself and its associated error variance. η_p_^2^ effects of 0.01 are seen as small, effects of 0.09 are evaluated as medium, and effects of 0.25 and higher are considered as large effects (48). Correlation analyses to test for associations between current BID features and eating/general psychopathology as well as weight (loss) were performed using Pearson product–moment correlation. Finally, a stepwise hierarchical logistic regression analysis was performed with sex and age (first step), psychopathological features such as disinhibited eating, depression, and anxiety (second step), and body image variables (third step) as predictors of successful weight loss. Analyses were performed separately for each treatment group.

## Results

### Sample Characteristics

Most of the participants were females (N = 205, 70.4%). There was no difference in the distribution of gender between the groups, whereas level of school education differed significantly between the groups, with the CONV group comprising the highest percentage of individuals with a higher-track school-leaving certificate. Moreover, as at baseline, there was a significant difference in age at the 9-year follow-up assessment: Individuals in the SURG group were significantly younger than those in the CONV group. Note that at the baseline assessment, there was a significant and large difference in body weight and BMI between all three groups, with the SURG group showing the highest BMI [means for BMI are displayed in **Table 1**, for more details see (30)]. Nine years after the start of the project weight and BMI did not differ between the treatment groups (SURG and CONV), but were still significantly higher in the treatment groups compared to the OC group. The SURG group achieved a significant and large weight loss over the 9-year period, compared to only marginal weight loss in the CONV group. OC participants slightly gained weight over the 9-year period. In total, about 41% of the SURG group met the definition of successful weight loss (%EBMIL still 50% or more)^1^ after the 9-year period, whereas 35% of the CONV group met the criterion of 5% weight loss maintenance.^2^ In the OC group, 28% reported 5% less weight compared to baseline weight. There was no significant difference in the number of successful weight loss (maintainers) between the groups. Details of these sample descriptions are presented in **Table 1**.

**Table 1:** Sample description.

		Group			Test statistics			
		SURG (N = 78)	CONV (N = 125)	OC (N = 83)	χ^2^/ANOVA	*p*	Ƞ^2^	Post-hoc/*p*
Gender (female)					χ^2^(2,284) = 0.924	0.630		
	N	54	91	56				
	%	69.2	73.4	67.5				
School education					χ^2^(6,269) = 24.061	0.001		
Low-tracker school diploma								
	N	30	30	25				
	%	38.5	25.8	30.1				
Middle-tracker school diploma								
	N	32	30	29				
	%	41	25.8	34.9				
High-tracker school diploma								
	N	10	55	23				
	%	12.8	44.4	27.7				
No school graduation								
	N	1	2	2				
	%	1.3	1.8	2.4				
Age (years)					*F*(2,285) = 4.528	<0.001	0.031	CONV>SURG
	M	46.19	50.78	49.45				0.009
	SD	9.59	10.88	11.11				
Weight baseline (kg)					*F*(2,285) = 9.350	<0.001	0.395	SURG<CONV>OC
	M	146.74	116.32	98.99				0.002/<0.001/<0.001
	SD	27.02	23.96	14.46				
Weight follow-up (kg)					*F*(2,285) = 9.350	0.012	0.062	SURG = CONV > OC
	M	113.85	114.84	100.27				0.002/ < 0.001
	SD	26.52	28.88	17.53				
BMI Baseline (kg/m^2^)					*F*(2,285) = 136.901	<0.001	0.493	SURG > CONV > OC
	M	50.41	39.76	34.27				< 0.001/ < 0.001/ < 0.001
	SD	7.15	6.86	4.23				
BMI follow-up (kg/m^2^)					*F*(2,285) = 10.535	<0.001	0.070	SURG = CONV > OC < 0.001/0.001
	M	39.27	39.13	34.69				
	SD	8.75	8.12	17.53				
Δkg					*F*(2,285) = 86.212	<0.001	0.379	SURG > CONV = OC < 0.001/ < 0.001
	M	32.97	1.48	−1.27				
	SD	25.31	17.97	9.86				
%WL					*F*(2,285) = 73.816	<0.001	0.344	SURG = CONV > OC
	M	21.42	1.18	−1.26				0.002/ < 0.001
	SD	15.0	14.02	9.73				
SWL					χ^2^(2,285) = 3.199	0.202		
	N	32	44	23				
	%	41	35	28				

SURG, bariatric surgery group; CONV, conventional treatment group; OC, obese control group. School education according to the German school system (Hauptschule, Realschule, Fachabitur/Abitur), BMI, body mass index; Δkg = baseline weight in kg minus actual weight in kg at 9-year follow-up assessment, note that positive scores represent weight loss, whereas negative scores indicate weight gain; %WL = percentage weight loss calculated as follows: [(baseline weight in kg − weight at 9-year follow-up in kg)/(baseline weight)] × 100; SWL, successful weight loss maintenance, indicates the number of individuals who did regain not more than 5% of the lost weight over the 9-year follow-up period or still report 50% of excess BMI loss (%EBMIL) in the SURG group respectively.

Regarding psychopathology at the 9 year follow-up, the largest number of individuals with an Axis I mental disorder were in the SURG group, followed by the CONV group and the OC group. However, the prevalence of binge eating disorder was rather low and did not differ between the groups. On the other hand, inhibition scores of the TFEQ subscale were significantly higher in the CONV group compared to the SURG and OC groups. Average depression and anxiety scores, assessed with the HADS, were below the cut-off and not of clinical relevance; however, both subscale scores were higher in SURG patients compared to CONV and OC patients. Detailed information regarding mean scores and standard deviations of the described characteristics as well as test statistics are displayed in **Table 2**.

**Table 2:** Psychopathology compared between groups.

		Group			Test statistics			
		SURG (N = 78)	*CONV* (N = 125)	OC (N = 83)				
					**χ^2^/ANOVA**	***p***	ɳ^2^	Post-hoc/*p*
Presence Axis I diagnoses					χ^2^(2,267) = 4.777	0.092		
	N	52	63	40				
	%	66.6	50.8	48.19				
BED					χ^2^(2,285) = 1.670	0.796		
	N	2	5	3				
	%	2.6	4.0	3.6				
Disinhibited eating (TFEQ)					*F*(2,285) = 15.688	<0.001	0.104	CONV > SURG = OC
	M	6.93	8.87	6.60				< 0.001/ < 0.001
	SD	3.83	3.42	3.95				
HADS—Depr					*F*(2,285) = 6.291	<0.001	0.044	SURG > OC
	M	6.55	5.35	4.19				0.001
	SD	4.98	3.75	3.88				
HADS—Anx					*F*(2,285) = 6.291	<0.001	0.034	SURG > OC
	M	7.47	6.61	5.42				0.006
	SD	4.48	4.23	3.35				

Diagnoses for Axis I disorders were assessed with the Composite International Diagnostic Interview (CIDI); BED, binge eating disorder, assessed with the structured interview for anorexic and bulimic disorders (SIAB); TFEQ, Three-Factor Eating Questionnaire; HADS, Hospital Anxiety and Depression Scale; Depr, depression subscale; Anx, Anxiety subscale.

### Perceptual Component of Body Image

The evaluation of the *perceived shape* using the BIA-O silhouettes revealed a significant time × group interaction: The perceived shape was largest in the SURG group for both baseline and current body image, but at the same time, changes in the perceived BI were only significant for this group (for details see **Table 3**). Both covariates, sex and age, exerted a significant influence on the estimation of the perceived body size, insofar as higher age reduced the effect and female gender increased it [*F*(2,270) = 11.804, *p* = 0.001, *η_p_^2^* = 0.042 and (*F*(2,270) = 12.402, *p* = 0.001, *η_p_^2^* = 0.044 respectively].

**Table 3:** Mean Scores and standard deviation for body image-related features.

Body image assessments			Group			Test statistics (for ANOVA interaction effects time × group)			
			SURG N = 77	CONV N = 118	OC N = 81	ANOVA	df	*p*	Ƞp^2^
Desired shape						1.557	2,271	0.273	
	Baseline score								
		M	7.03	6.27	6.11				
		SD	1.83	1.91	1.61				
	Current score								
		M	6.65	6.25	6.21				
		SD	2.02	1.78	1.63				
Perceived shape						39.435	2,270	<0.001	0.226
	Baseline score								
		M	15.05	11.51	9.28				
		SD	2.52	2.86	2.20				
	Current shape								
		M	10.55	11.09	9.47				
		SD	3.28	3.35	2.51				
BIAQ, 9 years ago									
	Clothes								
		M	5.86	6.79	4.38				
		SD	4.51	4.12	3.54				
	Social								
		M	2.22	1.46	1.33				
		SD	2.86	2.15	2.95				
	Eating control								
		M	3.45	3.72	3.36				
		SD	1.82	1.82	2.06				

Actual body shape and desired body shape were measured with the BIA-O (Body Image Assessment for Obesity); BIAQ, Body Image Avoidance Questionnaire.

### Attitudinal Component of Body Image

Regarding the *desired shape*, no significant time × group interaction emerged. However, a main effect of group [*F*(2,270) = 4.033, *p* = 0.019, *η_p_^2^* = 0.029] was found, indicating that SURG patients chose slightly larger silhouettes compared to CONV and OC. Moreover, there was a significant main effect of time [*F*(2,270) = 7.491, *p* = 0.007, *η_p_^2^* = 0.027] indicating a decrease over time above all in the SURG group. However, overall, at baseline, most of the participants chose a silhouette between 6 and 7, which corresponds to the higher range of normal weight/borderline overweight. Furthermore, the choice of the currently desired shape was also represented by the silhouettes numbered 6 and 7 (for details see **Table 3**). A significant effect emerged for sex as covariate ((*F*(2,270) = 5.746, *p* = 0.017, *η_p_^2^* = 0.021), indicating that overall, women chose more slender silhouettes than did men. Moreover, a significant time × age interaction emerged [*F*(1,271) = 8.872, *p* = 0.003, *η_p_^2^* = 0.032], with the chosen silhouettes becoming larger with increasing age.

**Figure 1:**
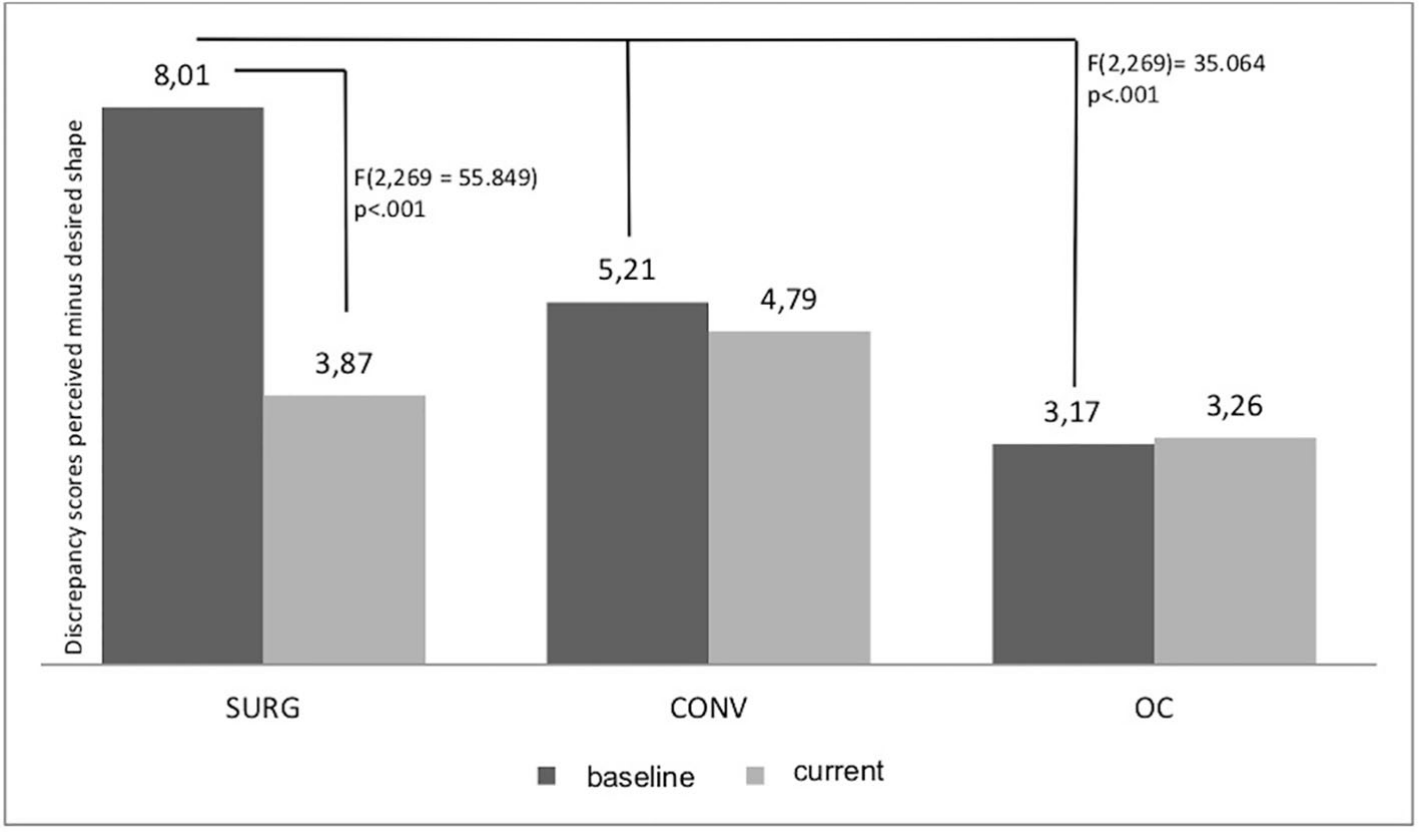
Discrepancy in current versus baseline body image dissatisfaction.

Regarding the degree of *body dissatisfaction* captured as the discrepancy score of current perceived shape estimation minus desired shape estimation, a significant time × group interaction emerged [*F*(2,269) = 55.849, *p* < 0.001, *η_p_^2^* = 0.293], indicating the highest improvement in the SURG group, compared to no changes in the OC and CONV group. We also found a main effect of group [*F*(2,269) = 35.064, *p* < 0.001, *η_p_^2^* = 0.207], such that SURG patients reported overall higher body dissatisfaction compared to OC and CONV. However, regarding only the current level of body dissatisfaction (at the 9-year follow-up) in individuals who had undergone bariatric surgery, body dissatisfaction was lower in SURG compared to CONV (*p* > 0.001) and comparable to that of OC (*p* = 1.0). Details are provided in **Figure 1**. Moreover, sex and age had a significant influence on the level of body dissatisfaction [main effects—sex: *F*(2,269) = 4.966, *p* = 0.027, *η_p_^2^* = 0.018; age: *F*(2,269) = 23.209, *p* < 0.001, *η_p_^2^* = 0.079]: Higher age was associated with lower dissatisfaction and female gender was associated with higher dissatisfaction.

### Behavioral Component of Body Image

A multivariate ANCOVA was performed to detect differences between the current level of body image avoidance between the three groups. For the subscale “clothing”, a significant difference between the groups emerged [*F*(2,285) = 8.597, *p* < 0.001, *η_p_^2^* = 0.058]. Sex as a covariate exerted a significant influence on avoidance behavior relating to clothing [*F*(2,285) = 7.680, *p* = 0.006], whereas age had no impact [*F*(2,285) = 2.230, *p* = 0.136]. Post hoc tests revealed that CONV reported higher avoidance behavior compared to OC (*p* < 0.001), whereas the level of behavioral avoidance did not differ between SURG and OC (*p* = 0.108) or between SURG and CONV (*p* = 0.251). For the subscales “social activities” and “eating control”, no significant difference between the groups emerged [*F*(2,285) = 2.658, *p* = 0.072 and *F*(2,285) = 0.653, *p* = 0.521 respectively]. For details, please see **Table 3**.

### Association of Body Image Features With Eating Psychopathology and General Psychopathology

The results of the correlation analyses revealed overall significant associations (all *p*s *≤*0.001) of both the current level of body dissatisfaction (BIA-O differences score) and the subscale score for clothing on the BIAQ with anxiety (*r_BIA-O_* = 0.200, *r_BIAQ_* = 0.320) and depression scores (*r_BIA-O_* = 0.380; *r_BIAQ_* = 0.533), eating disinhibition (*r_BIA-O_* = 0.441, *r_BIAQ_* = 0.468) as well as weight status (current BMI; *r_BIA-O_* = 0.650, *r_BIAQ_* = 0.354) and percentage of weight loss (*r_BIA-O_* = 0.650, *r_BIAQ_* = 0.354). Moreover, a moderate association was found for body dissatisfaction and avoidance behavior (*r* = 0.451).

### Impact of Body Image Features on Successful Weight Loss Maintenance

Finally, we employed hierarchical binary logistic regression analyses to categorize successful and unsuccessful weight loss maintenance separately for each group, in order to identify the impact of body image features on weight loss when controlling for the influence of eating disturbances and psychological features. Details of the final model (step 3 including all variables) for all three regression analyses are presented in **Table 4**.

**Table 4:** Third step regression analyses for weight loss maintenance.

Predictor	Group								
	SURG			CONV			OC		
	*β*	Wald	*p*	*β*	Wald	*p*	*β*	Wald	*p*
Sex	1.012	1.344	0.246	1.520	4.480	0.034	0.615	0.729	0.393
Age	−0.063	1.979	0.160	0.023	0.818	0.366	0.032	1.075	0.300
RSES	−0.130	0.617	0.432	−0.082	0.490	0.484	−0.006	0.002	0.966
TFEQ	0.044	0.180	0.671	−0.100	1.220	0.269	−0.197	3.610	0.057
HADS—Anx	0.234	4.046	0.044	−0.038	0.175	0.675	0.181	2.190	0.139
HADS—Depr	−0.224	2.471	0.116	−0.119	0.982	0.322	0.007	0.003	0.960
BIAQ_clothes	−0.231	3.480	0.062	−0.049	0.290	0.590	−0.120	0.822	0.365
BIAQ_social	0.085	0.269	0.604	0.266	3.369	0.066	0.039	0.133	0.715
change_dissatisfaction	0.532	12.140	<0.001	0.619	15.743	<0.001	0.723	10.648	0.001

SURG, bariatric surgery group; CONV, conventional treatment group; OC, obese control group; RSES, Rosenberg Self-esteem Scale; TFEQ, Three-factor Eating Questionnaire; disinhibition subscale; HADS, Hospital Anxiety and Depression Scale; Anx, anxiety; Depr, depression; BIAQ, Body Image Avoidance Questionnaire; subscale clothing.

In the *SURG group*, sex and age were not significant predictors in the first step. When depression, anxiety, and disinhibition were added as control variables, 30.7% of the variance was explained, with 75% of cases correctly classified, based on anxiety as well as depression scores as significant predictors (*β* = 0.335, *Wald* = 10.164, *p* = 0.001 and *β* = −0.280, *Wald* = 5.349, *p* = 0.021). The final, third step (adding change in body dissatisfaction, avoidance behavior subscales clothes and social activities) increased the explained variance to 57.3%, with 86.1% correctly classified cases. Anxiety and change in body dissatisfaction emerged as significant predictors, whereas depression was no longer significant. Avoidance behavior (subscale clothing) failed to reach statistical significance. All other variables had no statistically significant influence.

In the *CONV group*, sex emerged as a significant predictor in the first step (*β* = 1.146, *Wald* = 4.948, *p* = 0.026), but only explained 8.6% of the variance and with only 65.2% of correctly classified cases. Adding psychopathological variables in the second step increased the explained variance to 24.7%, with 68.8% of correctly classified cases. Sex remained a significant predictor (*β* = 1.305, *Wald* = 5.330, *p* = 0.021), and disinhibition emerged as significant (*β* = −0.176, *Wald* = 5.421, *p* = 0.020). In the third step, the model explained 51.2% of the variance, with 78.6% of correctly classified cases. Again, sex remained a significant predictor, and change in body dissatisfaction also emerged as a significant predictor, whereas avoidance behavior (social activities) failed to reach statistical significance and disinhibition lost its significant impact.

In the *OC group*, in the first step, neither sex nor age was significant. Adding psychopathological variables in the second step increased the explained variance (24.7%) and lead to 70% of correctly classified cases based on disinhibition as the only significant predictor. Anxiety was only of marginal statistical significance. In the third step, body image variables were entered. This final model explained 42.1% of the variance, and 85% of cases were correctly classified; here, change in body dissatisfaction was the only significant predictor, whereas disinhibition lost its statistical significance when change in body dissatisfaction was entered in the final step of the regression.

## Discussion

The main aim of this article was to explore the associations of different body image facets with weight loss outcome after bariatric surgery, and to compare these findings with conventional weight loss treatment outcomes and weight status among individuals with obesity who initially did not seek weight loss treatment. The data were derived from a larger prospective study, but body image assessment was captured solely at the 9-year follow-up, both for the current body image and for the (retrospectively evaluated) body image at baseline. The main results of the analyses revealed that solely the SURG patients reported a difference between the baseline and the current body image, suggesting an improvement in body satisfaction. Moreover, SURG patients reported lower levels of body avoidance in relation to clothing compared to CONV. No significant difference emerged between SURG and OC. The current level of body dissatisfaction as well as body avoidance behavior were associated with a range of psychopathological features, e.g. higher levels of depression and anxiety as well as disinhibited eating, higher body weight, and less weight loss. Regarding the role of body image in successful weight loss and the maintenance thereof, a positive change from baseline to current levels of body dissatisfaction was significantly associated with successful weight loss and the maintenance thereof, independently of the type of treatment. Several aspects need to be discussed in light of these results.

### Body Perception

On average, SURG participants retrospectively reported a larger baseline silhouette compared to the current one, which seems to reflect their change in weight. The SURG group lost the most weight and was able to maintain a larger proportion of the lost weight over the 9-year follow-up period compared to the CONV group and the OC group. These findings are in line with recent meta-analyses and systematic reviews (1, 8, 49). Nevertheless, more than half of the SURG group did not achieve/maintain the possible excess weight loss and was therefore not classified as successful according to the recommended definitions. This might be due to the restrictive nature of bariatric surgery procedures. CONV and OC participants showed no differences in baseline and current weight, which might reflect the lack of significant difference between the two chosen silhouettes. These results point toward a valid and reliable recall of body image at baseline. However, it has to be considered that body size estimations may be biased. There is some empirical evidence suggesting misperceptions in body size depending on BMI. For example, Thaler and colleagues (50) showed that women with a higher BMI tend to overestimate their shape. However, there is also evidence that women with obesity perceive their own body much more accurately than do normal-weight (51) and overweight women (52).

### Body Image Attitudes

Regarding the desired shape, the chosen silhouettes were in the normal-weight to borderline overweight range in all patients [silhouettes 6 and 7; see (39, 53)], but slightly higher in the SURG group compared to the OC group. However, among all patients, there was a shift toward normal-weight silhouettes from retrospectively reported baseline to 9 years later in terms of the desired shape. In line with previous findings of higher body dissatisfaction among women compared to men (26, 54), women in the present study indicated generally smaller silhouettes for their desired shape compared to men. This may reflect the general sociocultural influence on body image (55) and the negative portrayal of overweight and obese bodies that has become almost normative in the media (56).

Given that body dissatisfaction to some degree results from the experienced discrepancy between one’s own weight and the weight that is idealized or desired to be achieved through weight reduction [e.g. (26, 55)], it is not surprising that the perceived shape differed largely from the desired shape among all participants. As individuals with obesity are confronted daily with anti-fat attitudes and experience discrimination due to their weight (56, 57), their desire to lose weight and achieve a normal weight is high. In most cases, despite high investments, this goal is not realistic, leading to a possible decline in well-being following initial weight loss. This is also supported by recent evidence that shows associations between emotional distress and body dissatisfaction as well as depressive symptoms (6, 18, 58). Moreover, it is also in line with the correlational results from the present study: Both components—body dissatisfaction as well as avoidance behavior—were related to deficits in other areas of mental health (e.g. depression, anxiety), emphasizing the impact of body image on psychological functioning. Moreover, our results in relation to body avoidance behaviors showed no group differences regarding social activity and eating control, but higher scores for the CONV group in relation to clothing, which was also associated with higher dissatisfaction. From eating disorder research, it is known that body avoidance might maintain the negative body image and serve an anxiolytic function [e.g. (10, 59)]. However, body avoidance did not impact weight loss outcome. Further research is warranted to better understand this component of body image in individuals with obesity.

### Body Dissatisfaction and Its Association With Weight Loss

With regard to body dissatisfaction, we found a significant difference from recalled baseline to current body dissatisfaction only in the SURG group. This might be associated with the achieved weight reduction. In the CONV and OC group, the level of dissatisfaction did not change, which might be due to the lack of significant weight reduction. However, as we did not include prospective data, we cannot draw any conclusions regarding the direction of the association. Further research is required in order to elucidate the interaction between body image and weight loss. What has not been considered so far is that more than 90% of these individuals with severe obesity suffer from negative consequences of the fast and massive weight loss: “Hanging” skin arises and sometimes causes infections, as well as other distressing and non-aesthetic conditions (60–62). Whereas a large proportion of bariatric surgery patients seek skin-contouring surgery, only a small number of them actually receive it due to substantial barriers such as non-refundable surgery costs [e.g. (61)]. Recently, it was shown that skin correction surgery reduces depressive symptoms and improves body image (58). Moreover, it was reported that individuals who underwent body-contouring surgery after bariatric surgery showed more positive body image features compared to those who did not (60). Thus, as we did not include questions relating to these aesthetic complications and the wish for skin correction surgery, we do not know how these factors may have impacted body image features and weight loss after surgery.

### Strengths and Limitations

A strength of the present study lies in the application of a weight-specific assessment tool for body image in individuals with obesity, which captures different facets of body image across different treatment approaches, as well as the addition of an instrument that reflects behavioral components of body image. A further strength is the long-term follow-up, which allowed us to capture body image in surgery patients after their weight had stabilized or possibly increased again. However, several limitations of the present study also need to be considered: 1) The instruments for body image were added to the original study at the 9-year follow-up. Consequently, the baseline evaluation of body perception, desired shape, and ideal shape had to be made retrospectively, which may have biased the reported results. 2) The BIA-O does not assess dissatisfaction with specific body parts or loose skin and may therefore lack to capture the real level of body dissatisfaction as it may not only depend on the silhouette, but also on other features such as hanging or loose skin. As we did not ask whether body-contouring surgeries due to hanging or loose skin were wished, were necessary or were performed, following bariatric surgery, we cannot be sure that this might have biased body image evaluations [e.g. (61)]. 3) The sample assessed at the 9-year follow-up did not include those participants who dropped out of the study, meaning that the results might not be representative for all of the study participants. 4) Moreover, the BIA-O assesses body dissatisfaction using a difference score, which is not without criticism (63). 5) The number of SURG patients that report successful weight loss was remarkably low also at the 1 year follow-up which might reflect the problems of restrictive procedures. However, as up to 60% reductions of the excess weight (EWL, calculated as kilograms of weight above BMI 25 kg/m² as excess weight) can be reached depending on the surgical procedure (49), the present results have to be considered with care. Further research should therefore take into account the surgical procedure when addressing body image in bariatric surgery. 6) Finally, the use of different definitions for successful weight loss might bias the results. Replication of the presented results in larger, prospective trials is necessary to further our understanding of body image preceding and following bariatric surgery.

## Conclusion

The present study adds to the existing evidence by emphasizing the role of the different components of body image after surgery in mental health features and course of weight. A robust relationship between BID and weight loss, independently of weight loss approach, is implied and suggests the need to acknowledge body image in the aftercare of bariatric surgery as well as in conventional treatment [e.g. (19, 20, 28)].

## Data Availability Statement

The datasets generated for this study will not be made publicly available. At the time of the investigation, it was not usual to share data sets publicly and therefore the informed consent did not include such a statement. Therefore, participants did not give their consent to share the data sets publicly. However, if there are any requests, we will try to be responsive.

## Ethics Statement

The studies involving human participants were reviewed and approved by Ethical Board of the Medical Faculty of the Ruhr-University Bochum. The patients/participants provided their written informed consent to participate in this study.

## Author Contributions

SH and TL conceived the study concept and design (9-year assessment), SH and TL supervised data collection (9-year assessment), TL analyzed data and drafted the article. All authors (SH, AM, MZ and TL) were involved in interpreting and discussing the results, revising the article critically for important intellectual content, and had final approval of the submitted and published versions.

## Funding

This work was supported by the Deutsche Forschungsgemeinschaft (German Research Council, DFG; He2665/2-1, He2665/2-2), the Ministry of Education and Research (BMBF) (01GV0601), and the Institute Danone for Nutrition, Munich, Germany.

## Conflict of Interest

The authors declare that the research was conducted in the absence of any commercial or financial relationships that could be construed as a potential conflict of interest.

## Footnotes

1 One year after surgery, N = 36 participants (46%) reached the criteria of 50% EBMIL.

2 After 1 year of CONV treatment, N = 95 participants met the 5% reduction criteria (77%). Further information on weight loss success after treatment is available in 36.
