# Supplementary material for: Body Image and Body Avoidance Nine Years After Bariatric Surgery and Conventional Weight Loss Treatment
Source: Front Psychiatry. 2020 Jan 14;10:945. doi: 10.3389/fpsyt.2019.00945 (PMC6971062; doi:10.3389/fpsyt.2019.00945)
Supplement: Supplementary file 1 [file Table_1.docx]

**Supplement Information on ideal shape**

Regarding the *ideal shape*, the ratings focused on silhouettes in the normal weight range (silhouettes 4 to 5) and were comparable between the groups (*F*(2, 270)=2.870, *p*=.058); no discrepancy emerged from recalled to actual evaluated ideal figure (*F*(2,270)=.956, *p*=.329). No interactions were found (*F*(2,270)=.623, *p*=.537) and sex and age as covariates did not exert a significant influence ((*F*(1, 270)=.251, *p*=.616 and *F*(2, 270)=1.199, *p*=.275 respectively); however, the ideal shape differed depending on sex (*F*(2,270)=10.275, *p*=.002), namely males reported marked larger silhouettes than women as ideal shape. For means and standard deviation see Table 3.

Table S1

Mean scores and standard deviations for ideal shape

|  | | | Group | | |  | Test statistics | | | | | | | |
| --- | --- | --- | --- | --- | --- | --- | --- | --- | --- | --- | --- | --- | --- | --- |
| Body image assessments | | | SURG | CONV | OV |  |  |  | |  | | |  | |
|  | | |  |  |  |  |  | | | | | | | |
| BIA-0 silhouettes | | |  |  |  |  | ANOVA interaction effects Time*Group | | | | | | | |
|  | | |  |  |  |  | ANOVA | | df | | p | Ƞp^2^ | | |
| Ideal shape | |  | N=76 | N=117 | N=81 |  | .623 | | 2,270 | | .537 | - | | |
|  | baseline score | |  |  |  |  |  | |  | |  |  | | |
|  |  | M | 5.34 | 4.81 | 5.17 |  |  | |  | |  |  | | |
|  |  | SD | 1.67 | 1.47 | 1.49 |  |  | |  | |  |  | | |
|  | current score | |  |  |  |  |  | |  | |  |  | |  |
|  |  | M | 5.42 | 4.89 | 5.13 |  |  | |  | |  |  | |  |
|  |  | SD | 1.64 | 1.51 | 1.46 |  |  | |  | |  |  | |  |
|  | |  |  |  |  |  |  | |  | |  |  | |  |

Note. Actual body shape and body shape wished to achieve were measured with the BIA-O (Body Image Assessment for Obesity); BIAQ, Body Image Avoidance Questionnaire.
